# Simultaneous Identification of Ten Bacterial Pathogens Using the Multiplex Ligation Reaction Based on the Probe Melting Curve Analysis

**DOI:** 10.1038/s41598-017-06348-z

**Published:** 2017-07-19

**Authors:** Yixiang Jiang, Lianhua He, Pingfang Wu, Xiaolu Shi, Min Jiang, Yinghui Li, Yiman Lin, Yaqun Qiu, Fang Bai, Yiqun Liao, Qingge Li, RenLi Zhang, Qinghua Hu

**Affiliations:** 1 Shenzhen Center for Disease Control and Prevention, Shenzhen, 518020 China; 2 School of Life Sciences, Shenzhen University, Shenzhen, 518000 Guangdong China; 3 School of Life Sciences, Xiamen University, Xiamen, 361005 Fujian Province China

## Abstract

Pathogenic *Vibrio* spp., *Aeromonas* spp. and *Plesiomonas shigelloides* are associated with human gastroenteritis and wound infections, as well as fish diseases. The comprehensive and accurate identification of these pathogens is crucial for the current public health. The present study describes the development of a multiplex assay for the simultaneous identification of ten bacterial pathogens in a single reaction by using a multiplex ligation reaction based on probe melting curve analysis (MLMA). The specificity for target genes was 100%, as assessed with a panel of 67 bacterial pathogens, which indicated no cross-reactions. The detection limit of this assay ranged from 0.8 × 10^7^ CFU/mL to 1.5 × 10^8^ CFU/mL at the pure bacterial culture level and from 0.1 ng to 1.0 ng at the DNA level. The MLMA assay was used to detect ten species of pathogens in 269 clinical and seafood samples, and for further validation, the results were compared with the conventional culture method. The results indicated greater than 90% sensitivity and 100% specificity for each bacterial pathogen tested, and the kappa correlation for all the pathogens ranged from 0.95 to 1.00. Overall, this assay is well suited for public health laboratories for its high throughput, accuracy, and low cost.

## Introduction

*Vibrio* spp. are gram-negative bacteria that usually appear as motile rods and are abundant in estuarine and marine environments; there are approximately 72 species of vibrios (www.bacterio.cict.fr)^1–3^ that require sodium chloride for survival. Among the numerous *Vibrio* spp., *V*. *vulnificus*, *V*. *parahaemolyticus*, *V*. *mimicus*, *V*. *fluvialis*, *V*. *alginolyticus*, and *V*. *cholerae* are routinely isolated from human, clinical, and environmental samples; these pathogens are primarily involved in causing gastrointestinal illnesses, septicemia, wound infections and, in certain severe cases, death^1, 4–6^. Moreover, vibriosis, caused by a group of bacteria in the genus *Vibrio*, has resulted in considerable economic losses in marine aquaculture^7^. *V*. *parahaemolyticus* and *V*. *vulnificus* are the two most common causes of seafood-associated illnesses and deaths due to the consumption of raw or undercooked shellfish or other seafood^8^. *V*. *cholerae* is the causative agent of the disease cholera and is generally acknowledged to be an agent used in bioterrorism^9^. To date, only the strains of serogroups O1 and O139 have been identified as being responsible for human epidemic and pandemic cholera^10^; non-O1 and non-O139 *V*. *cholerae* strains are associated with a relatively mild gastroenteritis, thus occasionally resulting in a cholera-like illness in some serotypes^11^. It is crucial to know whether *V*. *cholerae* isolates carry the toxin genes to determine whether an isolate could cause pandemic cholera. *V*. *mimicus*, *V*. *fluvialis*, and *V*. *alginolyticus* have all been isolated in cases of diarrhea and wound infection in humans and diseases in fish^6, 12, 13^. *Plesiomonas shigelloides* has been implicated as an agent of human gastroenteritis and was formerly included in the family *Vibrionaceae* ^14^; however, most microbiologists currently agree that *P*. *shigelloides* is better classified as a member of the family *Enterobacteriaceae*. *Aeromonas*, a member of the *Aeromonadaceae* family, is ubiquitous in aquatic environments and can cause gastroenteritis as well as soft tissue infection^15, 16^, the diseases and illnesses caused by *Vibrio* spp. in humans have made the accurate identification of these organisms critical to the maintenance of public health.

To diagnose the above pathogens, the conventional method requires bacterial culture in alkaline peptone water, biochemical tests, and slide agglutination assays with specific antisera. These methods are often time consuming (2 to 7 days) and laborious. Furthermore, the variability in the biochemical characteristics of the conventional method may lead to a more complex interpretation of the results. Moreover, the accuracy level of six commercially available systems for identifying members of the family *Vibrionaceae* cannot ensure that the results obtained in every trial will be accurate (the accuracy rates range from 63.9% to 80.9%)^17^. Currently, molecular diagnostic methods, particularly multiplex nucleic acid-based assays, have been the most effective approaches, owing to their high specificity, sensitivity, and detection. Current molecular methods include the multiplex Polymerase Chain reaction (PCR) and multiplex real-time PCR assays, which can simultaneously detect two to seven targets in one closed tube^4, 18, 19^, and the Luminex assay can simultaneously detect six bacterial pathogens and seven enteric viruses^20, 21^. Jie Liu *et al*. have reported a TaqMan Array Card system that can detect 19 enteropathogens, including viruses, bacteria, and parasites, in one reaction^20^. Furthermore, multiplex ligase-dependent probe amplification (MLPA) has achieved a much higher throughput of up to 40 targets detected simultaneously^22^. However, there are drawbacks to the abovementioned methods, including their cost and vulnerability to contamination, and their need for specialized training that may not be available to all laboratories. Furthermore, to date, no assay system is able to identify all the bacterial pathogens in one reaction. In this regard, the development of an efficient, rapid, and accurate molecular method that can identify bacterial pathogens in one assay would be a useful tool for clinical laboratories and for the surveillance and diagnosis of human infections.

To increase the number of target genes and to improve the accuracy of detecting co-infection with two or more bacterial pathogens, we developed a multiplex ligation reaction based on a probe melting curve analysis (MLMA) assay for simultaneous identification of ten species of bacteria. After the multiplex ligation reaction, a linear-after-the-exponential (LATE)-PCR was carried out with a real-time PCR instrument, by using the fluorophore and melting temperature (Tm) value as dual labels, and a melting curve analysis (MCA) yielded a unique Tm code by which a certain pathogenic bacterium could be identified^23^. In this study, the analytical and clinical performances of the assay, compared with the conventional method, were determined by the evaluation of 67 reference isolates and 269 clinical and seafood samples. Early detection can help physicians correctly identify and treat patients affected by these bacterial pathogens and aid epidemiologists in tracking the source of the pathogens as quickly as possible, thereby limiting the economic loss associated with infected seafood.

## Results

### Genes Detected by the MLMA Assay

**Figure 1:**
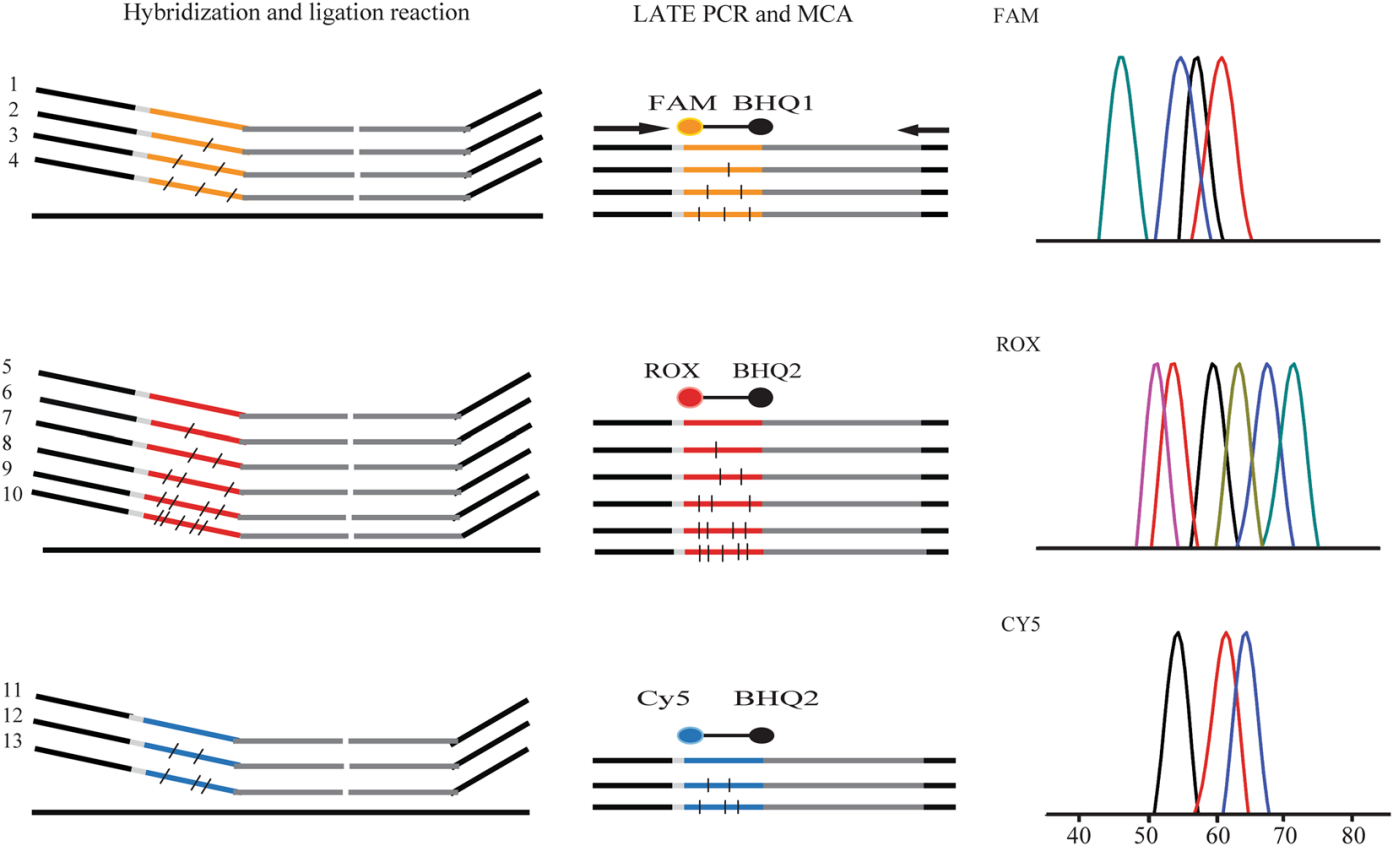
MLMA assay for the identification of ten bacterial pathogens in a single reaction. During the ligation process, 13 pairs of ligation oligonucleotides were hybridized onto the target genes of ten bacterial pathogens and one internal control (IC). In the LATE-PCR/MCA step, the ligated products were amplified by one pair of universal primers in the presence of three differently labeled fluorogenic probes; after PCR amplification, the unique Tm value was obtained via the MCA. FAM: IAC, *P*. *shigelloides*, *Aeromonas*, *V*. *parahaemolyticus* (*tdh*); ROX: *V*. *cholerae*, *V*. *fluvialis*, *V*. *vulnificus*, *V*. *parahaemolyticus*, *V*. *mimicus*, *V*. *alginolyticus;* Cy5: O1 *V*. *cholerae*, O139 *V*. *cholerae*, *V*. *cholerae* (*CTX*).

The MLMA assay is schematically illustrated in Fig. 1. During the detection of the ten bacterial pathogens, three probes that were fluorescently labeled with carboxyfluorescein (FAM), carboxy-X-rhodamine (ROX), and indodicarbocyanine-5 (Cy5) and 13 Tm tags (Supplementary Table S5) were used to hybridize the target DNA sequences to generate unique Tm values. In the FAM channel, *Aeromonas*, *Plesiomonas shigelloides*, the virulence gene thermostable direct hemolysin (*tdh*), and an internal control (IC) gene were detected. In the ROX channel, *V*. *cholera*, *V*. *fluvialis*, *V*. *vulnificus*, *V*. *parahaemolyticus*, *V*. *mimicus*, and *V*. *alginolyticus* were detected. In the CY5 channel, O1 *V*. *cholera*, O139 *V*. *cholera*, and the virulence gene *CTX* were detected. Thirteen specific genes (including an *SUC2* gene) were developed for identification of the pathogenic vibrios, particularly *V*. *cholera* and *V*. *parahaemolyticus*. The MLMA assay not only simultaneously distinguished the O1 and O139 strains from non-O1 and non-O139 strains but also was capable of determining whether the detected bacteria had the toxin gene.

### Evaluation of the Analytical Performance of the MLMA Assay

**Figure 2:**
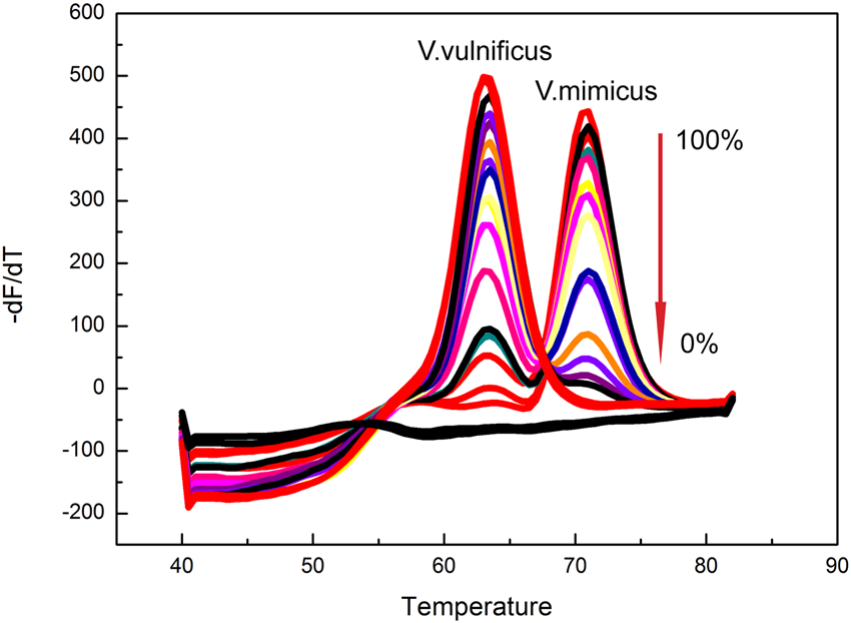
Detection results of the mixed infection simulation. *V*. *vulnificus* and *V*. *mimicus* were simultaneously detected and verified to co-exist in one sample in the same channel. Melting curves of artificial templates consisting of two vibrios with various percentage ratios (0:100, 1:99, 3:97, 5:95, 10:90, 20:80, 30:70, 40:60, 50:50, 60:40, 70:30, 80:20, 90:10, 95:5, 97:3, 99:1 to 100:0) were tested. The overall template concentration was 10^8^ copies per reaction. DEPC water was used because there were no template controls.

The newly developed MLMA assay exhibited high specificity (Fig. 2). No cross-reaction was observed when the reference strains were tested (Supplementary Table S1). The assay was able to detect all the target genes and yielded the expected Tm values (Supplementary Table S5), thus indicating that the developed assay has significant specificity.

The reproducibility of this MLMA assay was evaluated by detecting two ten-fold serial dilutions (3 × 10^10^ and 3 × 10^8^ copies/mL) in triplicate. The intra-assay and inter-assay standard deviations (SDs) and coefficient of variation (CV) values were calculated and are listed in Table 1. The largest SD value was no more than 1 °C, thus indicating that the assay was highly reproducible. The intra-assay CVs for each vibrio ranged from 0 to 2%, and the inter-assay CVs ranged from 0 to 1%. The limits of detection (LODs) were determined to be 0.1 ng to 1.0 ng at the DNA level and ranged from 0.8 × 10^7^ CFU/mL to 1.5 × 10^8^ CFU/mL at the bacterial level (Table 2).

**Table 1:** Reproducibility of the MLMA assay.

Type	Gene	Concentration	Intra-assay reproducibility			Inter-assay reproducibility		
		(Bacilli/mL)	Mean Tm (°C)	SD	CV(%)	Mean Tm (°C)	SD	CV(%)
*V*. *cholerae*	*hlyA*	3 × 10^10^	53.0	0.00	0.00	52.8	0.29	0.01
		3 × 10^8^	53.0	0.00	0.00	52.8	0.29	0.01
	*CTX*	3 × 10^10^	68.3	0.00	0.00	68.5	0.50	0.01
		3 × 10^8^	68.5	0.00	0.00	68.2	0.29	0.01
	O1*rbf*	3 × 10^10^	54.8	0.29	0.01	54.8	0.29	0.01
		3 × 10^8^	54.8	0.29	0.01	54.8	0.29	0.01
	O139*wbfR*	3 × 10^10^	61.7	0.29	0.00	61.8	0.58	0.01
		3 × 10^8^	62.0	0.00	0.00	62.0	0.00	0.00
*V*. *fluvialis*	*toxR*	3 × 10^10^	55.5	0.00	0.00	55.2	0.29	0.01
		3 × 10^8^	55.0	0.00	0.00	55.2	0.29	0.01
*V*. *vulnificus*	*vvh*	3 × 10^10^	61.5	0.00	0.00	61.5	0.00	0.00
		3 × 10^8^	61.7	0.29	0.00	61.7	0.29	0.00
*V*. *parahaemolyticus*	*toxR*	3 × 10^10^	65.0	0.00	0.00	65.2	0.29	0.00
		3 × 10^8^	65.0	0.00	0.00	65.0	0.00	0.00
	*tdh*	3 × 10^10^	63.2	0.29	0.00	63.0	0.50	0.00
		3 × 10^8^	63.0	0.00	0.00	62.8	0.29	0.00
*V*. *mimicus*	*vvh*	3 × 10^10^	69.5	0.00	0.00	69.5	0.00	0.00
		3 × 10^8^	69.5	0.00	0.00	69.5	0.00	0.00
*V*. *alginolyticus*	*Collagenase*	3 × 10^10^	73.0	0.00	0.00	72.8	0.29	0.00
		3 × 10^8^	73.2	0.29	0.00	73.2	0.29	0.00
*P*. *shigelloides*	*23S rRNA*	3 × 10^10^	57.5	0.87	0.02	57.0	0.50	0.01
		3 × 10^8^	57.2	0.29	0.01	56.8	0.29	0.01
*Aeromonas*	*16S rRNA*	3 × 10^10^	59.7	0.29	0.00	59.3	0.29	0.00
		3 × 10^8^	59.5	0.00	0.00	59.2	0.29	0.00

**Table 2:** The detection limit of the MLMA assay at both the DNA and bacterial levels.

Type	Gene	LOD	
		DNA level (ng/μL)	Bacterial level (CFU/mL)
*V*. *cholerae*	*hlyA*	0.1	0.8 × 10^7^
	*CTX*	1.0	1.0 × 10^7^
O1 *V*. *cholerae*	O1*-rfbE*	1.0	1.2 × 10^8^
O139 *V*. *cholerae*	O139*-wbfR*	1.0	1.5 × 10^8^
*V*. *fluvialis*	*toxR*	0.1	0.9 × 10^7^
*V*. *vulnificus*	*vvh*	0.1	0.7 × 10^7^
*V*. *parahaemolyticus*	*toxR*	0.1	1.4 × 10^7^
	*tdh*	0.1	1.4 × 10^7^
*V*. *mimicus*	*vvh*	0.1	0.9 × 10^7^
*V*. *alginolyticus*	*Collagenase*	0.1	1.1 × 10^7^
*P*. *shigelloides*	23*S rRNA*	0.1	1.2 × 10^8^
*Aeromonas*	16*S rRNA*	0.1	1.5 × 10^8^

**Figure 3:**
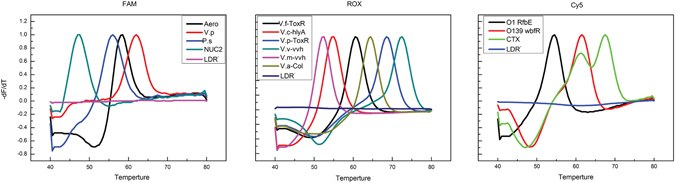
Specificity of the MLMA assay in detecting reference strains from different sources.

Taking into account the co-infection in stool samples or seafood samples, two bacteria, either present simultaneously in the same fluorescence channel or present in two different fluorescence channels, were selected to simulate mixed infection by varying the relative ratios of the bacteria. The results (Fig. 3 and Supplementary S7) showed that the lowest level of one species of bacteria that could be detected was 3% in the presence of 97% of the other type of bacteria, thus indicating that the MLMA assay can detect mixed infections over a wide range.

### Clinical Studies

A total of 269 clinical and seafood samples were tested with the MLMA assay, and the results were compared with those using the conventional method (Table 3). Additionally, four singleplex qPCR assays targeting the virulence gene for *V*. *cholera* and *V*. *parahaemolyticus* (Supplementary Table S4) were performed in parallel. All the samples that were positive for *V*. *cholerae*, *V*. *fluvialis*, *V*. *vulnificus*, *V*. *parahaemolyticus*, *V*. *mimicus*, *V*. *alginolyticus*, *P*. *shigelloides*, and *Aeromonas* according to the MLMA assay were also examined simultaneously through conventional methods, and the detection results for the virulence gene for *V*. *cholera* and *V*. *parahaemolyticus* were also consistent with the MLMA assay results. Among the 145 *V*. *parahaemolyticus-*positive samples, 91 tested positive for the *tdh* gene, and among the 62 *V*. *cholera-*positive samples, 41 tested positive for the *CTX* gene (Supplementary Table S8).

**Table 3:** Performance of the MLMA assay compared with the conventional method.

MLMA assay	Conventional method		Sensitivity	Specificity	Agreement	Kappa Value
	Positive	Negative	(%)	(%)	(%)	
Non-O1/O139 *V*. *cholerae*						
Positive	17	0	100.0	100.0	100.0	1.00
Negative	0	252				
O1 *V*. *cholerae*						
Positive	43	0	100.0	100.0	100.0	1.00
Negative	0	226				
O139 *V*. *cholerae*						
Positive	2	0	100.0	100.0	100.0	1.00
Negative	0	267				
*V*. *fluvialis*						
Positive	14	1	93.3	100.0	99.6	0.96
Negative	0	254				
*V*. *vulnificus*						
Positive	20	0	100.0	100.0	100.0	1.00
Negative	0	249				
*V*. *parahaemolyticus*						
Positive	139	6	95.8	100.0	97.7	0.95
Negative	0	124				
*V*. *mimicus*						
Positive	1	0	100.0	100.0	100.0	1.00
Negative	0	268				
*V*. *alginolyticus*						
Positive	66	0	100.0	100.0	100.0	1.00
Negative	0	203				
*P*. *shigelloides*						
Positive	4	0	100.0	100.0	100.0	1.00
Negative	0	245				
*Aeromonas*						
Positive	11	0	100.0	100.0	100.0	1.00
Negative	0	258				

In addition, 54 samples were identified as being co-infected with *V*. *parahaemolyticus* and *V*. *alginolyticus*. However, with the conventional methods, only 48 samples were found to be positive for multiple pathogens. Most of the discrepancies (6/54) were MLMA-po-sitive/conventional-method-negative results, and the probable reason for the discrepant results (6/54) between the two methods was that the results that were MLMA positive but co-nventional method negative often had lower fluorescence intensities, because a lower bacterial concentration could not be isolated through the conventional method. The qPCR results appeared to support the MLMA results (Supplementary Table S6).

Overall, according to the detection results of the 269 clinical and seafood samples, the sensitivities of the MLMA assay versus the conventional method were 100% (*V*. *cholerae*), 93.3% (*V*. *fluvialis*), 100% (*V*. *vulnificus*), 95.8% (*V*. *parahaemolyticus*), 100% (*V*. *mimicus*), 100% (*V*. *alginolyticus*), 100% (*P*. *shigelloides*) and 100% (*Aeromonas*), and the specificities were 100%. The concordance between the MLMA assay and conventional method results for all the pathogens was >97%, and the kappa values ranged from 0.95 to 1.0 (Table 3).

## Discussion

It has long been established that the pathogenic *Vibrio* spp., *Aeromonas* and *Plesiomonas shigelloides* not only are associated with human diarrhea, septicemia, and wound infections but also affect the seafood industry^24–26^. In developing countries, *Vibrio* infections occur more frequently, owing to the lack of safe drinking water and the consumption of raw seafood^27^. Therefore, the accurate identification of these high-risk organisms is essential.

We successfully developed an MLMA assay for the simultaneous identification of ten pathogenic bacteria in clinical samples and seafood in a single reaction tube. To our knowledge, the MLMA assay is the first to identify these bacterial pathogens in a single reaction, and thus this method has extensive application prospects. All the target genes were accurately identified, and no cross-reaction was observed, even in the presence of genetically related bacterial species. The LOD for each gene was 0.8 × 10^7^ CFU/mL to 1.5 × 10^8^ CFU/mL at the level of pure bacterial cultures and 0.1 ng to 1 ng at the genomic DNA level, and the reproducibility was 100% in all aspects. Moreover, this method accurately detected multiple pathogens in one sample over a broad range. The MLMA assay displayed greater than 90% sensitivity and 100% specificity for each pathogen, and the kappa correlation values for the detection of all the pathogens ranged from 0.95 to 1.00.

An increasing number of recently published studies have focused on the development of molecular methods to solve multiple detection in one reaction^24, 28–30^. Recently, MCA has been used to achieve a higher throughput assay for multiplex detection^31, 32^. The MLMA assay developed and described in this study combined MLPA, LATE-PCR, and MCA techniques to achieve multiplex nucleic acid detection. MLMA and MLPA differ in their respective detection methods. MLPA detects target genes through a single capillary electrophoresis, in which each probe gives rise to an amplification product of a unique size between 130 and 480 bp^22^, whereas MLMA uses MCA to detect the target DNA sequence with a real-time PCR instrument, thus yielding unique Tm values^23^. A universal primer amplifies all the ligated products after the ligation reaction. Then, three detection probes hybridize with the artificial sequences contained in the ligated product, thus generating a unique Tm value through MCA in the FAM, ROX, and Cy5 fluorogenic channels. The MLMA assay, by allowing these bacterial pathogens to be identified in a single reaction, has broad application prospects.

The MLMA assay has three advantages, as compared with the existing methods. First, a detection probe is used to hybridize all the target sequences in one channel. A real-time PCR instrument with four fluorescence channels can distinguish at least 24 target genes through MCA. Second, compared with currently available methods, such as the conventional method or a multiplex PCR assay, which may lead to ambiguous results, the results of the MLMA assay can be automatically and accurately obtained within 3 h. Third, the MLMA assay is extremely cost-effective; the cost of a fluorescence detection method primarily depends on the consumption of the fluorescence probe. For example, if 15 target genes require detection, three fluorescence probes can be used in the MLMA assay, whereas at least 18 fluorescence probes are required to complete the same task by using multiplex real-time PCR. Therefore, the MLMA assay is very cost-effective.

Another distinct advantage of our MLMA assay is that this method can detect a mixed infection in one sample. Many studies have reported the occurrence of mixed infections in clinical and seafood samples with a small proportions^33, 34^. Understanding the significance of a mixed infection may help direct the development of treatment strategies. However, the conventional method often depends on the expertise of the operator when single colonies are extracted from plates, and therefore, only the dominant pathogen may be identified, while the minor and other suspicious colonies are frequently overlooked. In this study, the reason of the discrepant results (6/54) between the two methods may have been that minor single colonies in portions of the samples were ignored or not cultured. In our previous study^35^, we have used the multicolor combinational probe coding (MCPC) strategy to develop a multiplex assay for the simultaneous detection of five enteric viruses. Although this approach can achieve rapid detection, it is still difficult to determine whether a result indicates a mixed infection if both the FAM and HEX fluorescence signals from dual labeling appear with a high Ct value. Other current methods, such as DNA sequencing or restriction fragment length polymorphism, also focus on the dominant pathogen of the sample. However, our MLMA assay was able to identify a pathogen within a mixed infection with an abundance as low as 3% in the presence of the other pathogen. The consistency between the results of this method and those of singleplex rt-PCR fully illustrates the ability of the MLMA assay to distinguish mixed infections.

Notably, there are several limitations of the MLMA assay that require improvement. First, this method cannot be used for direct detection because the low LOD requires the sample to be cultured overnight before detection; therefore, the MLMA assay is more suitable for identification than for rapid detection. Second, compared with real-time PCR detection, the MLMA assay involves more complicated procedures in the ligation step. Therefore, further studies should address combining the ligation reaction with PCR amplification and MCA. Third, an insufficient number of *V*. *mimicus* strains were used to conclude significant evaluations regarding sensitivity and specificity; therefore, more samples will be collected for further validation in the future.

In conclusion, the MLMA assay developed for the accurate identification of these ten bacterial pathogens differs from established molecular diagnostic methods in that it is high throughput, less costly, contamination-free, and easier to use. Furthermore, this strategy can be modified and extended to detect more pathogens associated with human diseases in clinical settings. This assay is a sensitive and specific tool for the simultaneous identification of the major bacterial pathogens causing gastroenteritis and wound infections in humans and diseases in fish.

## Materials and Methods

### Bacterial Strains and Genomic DNA Preparation

The present study used 67 bacterial isolates for the initial development and accuracy evaluation of the MLMA assay, including 51 reference strains from the American Type Culture Collection (ATCC) and the National Center for Medical Culture Collections of China (CMCC), and 16 positive clinical isolates (see Table S1 in the supplemental material). These isolates were obtained from Shenzhen Center for Disease Control and Prevention (Shenzhen CDC, Shenzhen, Guangdong Province, China) and Chinese Center for Disease Control and Prevention (China CDC, Changping, Beijing, China). Genomic DNA was isolate-d using a QIAamp DNA Mini Kit (QIA-GEN; Hilden, Germany), according to the manufacturer’s protocol. The isolated DNA was quantified using a Nanodrop spectrophotometer (N-anodrop Products, Wilmington, Delaware, USA). Prepared genomic DNA was stored at −2-0 °C for use.

### Preparation of Clinical Isolate DNA

A total of 269 clinical specimens and seafood samples were collected from the Shenzhen Center for Disease Control and Prevention (Shenzhen CDC, Shenzhen, Guangdong Province, China). All the samples were first incubated with alkaline peptone water (APW [pH 8.6]; 3% NaCl) for 12 h at 37 °C. The previously described simple heating lysis method was used for DNA extraction from 1 mL of sample culture medium^19^. The DNA samples were analyzed using the MLMA assay, which is described in the subsequent section.

### Procedures for Conducting the MLMA

In general, the MLMA assay system includes two steps: (1) Hybridization and ligation; and (2) PCR amplification and melt curve analysis. During the hybridization-ligation process, each probe consisted of two oligonucleotides that hybridized at adjacent sites of the target sequence in the DNA sample, and these oligonucleotides were then ligated with DNA ligase after hybridization. A universal PCR primer pair was designed to amplify the ligated product via LATE-PCR by using a standard real-time PCR instrument. During the detection process, an MCA was performed for each sample after the PCR amplification to detect the target DNA sequence, thus resulting in unique Tm values displayed by the hybrids formed between the Tm tags and their corresponding fluorescent probes in the respective fluorophore channels. Finally, the identification results for each pathogen were determined on the basis of the Tm^23^.

Step 1: Hybridization and Ligation

The hybridization-ligation reaction was performed with a T3 Thermocycler (Biometra, Germany) and 1.5 μL of ligation probe mix (1–4 fmol of each specific ligation oligonucleotide, including an IC, Supplementary Table S2). A 5-μl volume of the previously isolated genomic DNA was denatured at 98 °C for 5 min and allowed to reach 75 °C, and finally, a 3.5-μL reaction mixture containing 1 μL of DNA ligase buffer, 1 unit of Taq DNA ligase (New England Biolabs, Beijing, China), and 1.5 μL of diethylpyrocarbonate (DEPC) water was added to the reaction tube. The samples were then incubated at 60 °C for 80 min, 95 °C for 5 min, and finally cooled to 4 °C.

Step 2: PCR Amplification and MCA

The reactions for amplification and MCA were performed on a Bio-Rad CFX 96 real-time PCR system (Bio-Rad Inc., Hercules, CA). The reaction mixture (50 μL) contained 1 × PCR buffer, 3 mM MgCl_2_, 2 μL of deoxynucleoside triphosphate (2.5 mM), 1 unit of Taq polymerase, 0.015 μM limiting primer, 0.4 μM excess primer, 0.08 μM to 0.16 μM fluorogenic probes (Supplementary Table 3), and 5 μL of ligation product from step 1. The amplification procedure consisted of an initial denaturation at 95 °C for 3 min, followed by 40 cycles of 95 °C for 5 s, 56 °C for 15 s, and then 74 °C for 15 s. Then, the MCA began with denaturation for 2 min at 95 °C, hybridization for 2 min at 40 °C, and a stepwise temperature increase (1 °C per 5 s) from 40 °C to 85 °C. The levels of FAM, ROX, and Cy5 fluorescence were collected and recorded during the MCA procedure.

### Evaluation of the Analytical Performance of the MLMA Assay

#### Assessment of the Specificity of the MLMA Assay

Evaluation of the analytical specificity was performed by assessing DNA lysates prepared from pure cultures of 67 reference strains obtained from different sources (Supplementary Table 2), according to the procedures of the MLMA assay and comparison of the results with traditional culture-based methods.

#### Measurement of the LOD and Reproducibility of the MLMA Assay

The LOD was determined by using both a series of 10-fold dilutions of the purified genomic DNA from 10 ng to 0.01 ng and lysates obtained from the reference strains starting from 10^10^ to 10^5^ CFU/mL. A turbidity meter was used for estimation.

Two ten-fold serial dilutions (3 × 10^10^ and 3 × 10^8^ copies/mL) were prepared to analyze reproducibility.

DNA extraction was conducted by using a QIAamp DNA Mini Kit (QIAGEN, Hilden, Germany). Each concentration was detected in triplicate with the MLMA assay.

#### MLMA Assay Detection of a Mixed Infection

Both clinical samples and seafood samples often contain mixed infections. Therefore, to increase the relevance of the study results to real world situations, in the same fluorescence channel, *V*. *mimicus* and *V*. *vulnificus*, *V*. *vulnificus* and *V*. *alginolyticus*, *V*. *parahaemolyticus* and *V*. *alginolyticus*, *V*. *fluvialis* and *V*. *vulnificus*, as well as *V*. *cholera* and *V*. *vulnificus* were used as examples to simulate co-infection. In different fluorescence channels, *V*. *cholera* and *P*. *shigelloides*, as well as *V*. *mimicus* and *Aeromonas*, were used as examples to simulate co-infection. The MLMA assay was performed with various percentage ratios of the two species (100:0, 97:3, 95:5, 90:10, 80:20, 70:30, 60:40, 50:50, 40:60, 30:70, 20:80, 10:90, 5:95, 3:97, and 0:100), and DEPC water was used as a negative control.

### Verification of Multiplex Results through Singleplex Real-Time PCR Reactions

To verify the multiplex results, four rt-PCR reactions were carried out to detect *V*. *cholera*, *CTX*, *V*. *parahaemolyticus*, and the *tdh* gene of *V*. *parahaemolyticus* (Supplementary Table S4) by using a CFX96 real-time system (Bio-Rad) in a 25-μl reaction mixture. The PCR components were 1 × PCR buffer, 3 mM MgCl_2_, 2 mM dNTP, 1 U/μL Taq polymerase, 10 pmol each of forward and reverse primers, 4 pmol of TaqMan probe, and 5 μL of DNA template under the following conditions: 95 °C for 3 min, followed by 40 cycles of 95 °C for 10 s, 55 °C for 30 s, and 72 °C for 20 s, and the fluorescence intensity in the FAM detection channel was recorded.

### Clinical Specimens and Statistical Analysis

A double-blind test was performed for all samples to evaluate the performance of the MLMA assay compared with the conventional method. A total of 269 clinical specimens and seafood samples were enriched in parallel with pathogen-specific enrichment broth. Then, a loopful of bacteria from each enrichment culture was streaked onto appropriate agar plates, and a minimum of five typical colonies were selected and subjected to biochemical and serological tests for identification according to the Chinese National Food Safety Standard “Food microbiological examination” (GB4789-2010, China).

The results of the multiplex rt-PCR assay were compared with those of the traditional culture-based methods by using a 2 × 2 table to estimate indices of sensitivity and specificity. The kappa correlation was also calculated.

## Electronic supplementary material

The supplementary materials of the MLMA assay

The detection results of the 269 seafood and clinical samples
