# Supplementary material for: Simultaneous Identification of Ten Bacterial Pathogens Using the Multiplex Ligation Reaction Based on the Probe Melting Curve Analysis
Source: Sci Rep. 2017 Jul 19;7:5902. doi: 10.1038/s41598-017-06348-z (PMC5517457; doi:10.1038/s41598-017-06348-z)
Supplement: Supplementary file 1 — The supplementary materials of the MLMA assay [file 41598_2017_6348_MOESM1_ESM.pdf]

Simultaneous Identification of Ten Bacterial Pathogens Using the Multiplex Ligation Reaction Based on the Probe Melting Curve Analysis

Yixiang Jiang[1], Lianhua He[1], Pingfang Wu[1], Xiaolu Shi[1], Min Jiang[1], Yinghui Li[1], Yiman Lin[1], Yaqun Qiu[1], Fang Bai[2],Yiqun Liao[3], Qingge Li[3], RenLi Zhang[1], Qinghua Hu[1,2 +]

Affiliation:

[1]Shenzhen Center for Disease Control and Prevention, Shenzhen 518020, China

[2]School of Life Sciences, Shenzhen University, Shenzhen 518000, Guangdong, China

[3]School of Life Sciences, Xiamen University, Xiamen 361005, Fujian Province, China

Supplementary Information:

The supplementary materials of the MLMA assay including reference strains, primer and probe sequences, the discrepant results and the detection results of mixed infection

Table S1 Reference strains used in study

Reference strains	Number	Source	Type
Acinetobacter baumannii	1	Shenzhen CDC	ATCC 19606
Bacillus cereus	1	Shenzhen CDC	ATCC 11778
Campylobacter jejuni	1	Shenzhen CDC	ATCC 33292
Enterobacter aerogenes	1	Shenzhen CDC	CMCC 45103
Enterobacter cloacae	1	Shenzhen CDC	CMCC 45301
Enterococcus faecalis	1	Shenzhen CDC	ATCC 29212
Enterobacter sakazakii	1	Shenzhen CDC	ATCC 51329
Klebsiella pneumoniae	1	Shenzhen CDC	ATCC 700603
Listeria monocytogenes	1	Shenzhen CDC	ATCC 19114
Proteus mirabilis	1	Shenzhen CDC	ATCC 29906
Proteus vulgaris	1	Shenzhen CDC	CMCC 49001
Pseudomonas aeruginosa	1	Shenzhen CDC	ATCC 27853
Shigella boydii	1	Shenzhen CDC	CMCC 51265
Shigella dysenteriae	1	Shenzhen CDC	CMCC 51376
Shigella flexneri	1	Shenzhen CDC	ATCC12022
Shigella sonnei	1	Shenzhen CDC	CMCC 51081
Staphylococcus aureus	1	Shenzhen CDC	ATCC 27664
Streptococcus hemolytic-β	1	Shenzhen CDC	CMCC 32210
Salmonella aberdeen	1	Shenzhen CDC	CMCC 50313
Salmonella abortus-equi	1	Shenzhen CDC	CMCC 47717
Salmonella adelaide	1	Shenzhen CDC	CMCC 50065
Salmonella anatum	1	Shenzhen CDC	CMCC 50083
Salmonella cholerae-suis	1	Shenzhen CDC	CMCC 47649
Salmonella coli	1	Shenzhen CDC	CMCC 50809
Salmonella dublin	1	Shenzhen CDC	CMCC 50042
Salmonella enteritidis	1	Shenzhen CDC	CMCC 50040
Salmonella gallinarum	1	Shenzhen CDC	CMCC 50770
Salmonella infantis	1	Shenzhen CDC	CMCC 50341
Salmonella javiana	1	Shenzhen CDC	CMCC 50364
Salmonella kentucky	1	Shenzhen CDC	CMCC 50794
Salmonella litchfield	1	Shenzhen CDC	CMCC 50810
Salmonella london	1	Shenzhen CDC	CMCC 50310
Salmonella Manchester	1	Shenzhen CDC	CMCC 50380
Salmonella manhattan	1	Shenzhen CDC	CMCC 50151
Salmonella meleagridis	1	Shenzhen CDC	CMCC 50329
Salmonella minnesota	1	Shenzhen CDC	CMCC 50061
Salmonella moscow	1	Shenzhen CDC	CMCC 50044
Salmonella muenchen	1	Shenzhen CDC	CMCC 50125
Salmonella newport	1	Shenzhen CDC	CMCC 50029
Salmonella oranienburg	1	Shenzhen CDC	CMCC 50379
Salmonella paratyphi A	1	Shenzhen CDC	CMCC 50001

Salmonella paratyphi B	1	Shenzhen CDC	CMCC 50004
Salmonella paratyphi C	1	Shenzhen CDC	CMCC 50017
Salmonella typhi	1	Shenzhen CDC	CMCC 50097
Salmonella typhi-suis	1	Shenzhen CDC	CMCC 50734
Salmonella typhimurium	1	Shenzhen CDC	CMCC 50013
Vibrio cholerae	1	Shenzhen CDC	ATCC 14101
O1 Vibrio cholerae	1	Shenzhen CDC	clinicai isolates
O139 Vibrio cholerae	1	Shenzhen CDC	clinicai isolates
Vibrio parahaemolyticus	1	Shenzhen CDC	ATCC17082
Vibrio parahaemolyticus (tdh+)	2	Shenzhen CDC	clinicai isolates
Vibrio vulnificus	1	Guangdong CDC	ATCC 27562
Vibrio mimicus	1	China CDC	clinicai isolates
Vibrio fluvialis	1	China CDC	clinicai isolates
Vibrio alginolyticus	3	China CDC	clinicai isolates
Plesiomons shigelloides	1	Shenzhen CDC	clinicai isolates
Aeromonas	3	Shenzhen CDC	clinicai isolates
Enterotoxigenic E.coli	1	Shenzhen CDC	clinicai isolates
Enteroaggregative E.coli	1	Shenzhen CDC	clinicai isolates
Enteroinvasive E.coli	1	Shenzhen CDC	clinicai isolates
Enterohemolysin E.coli	1	Shenzhen CDC	clinicai isolates
Enteropathogenic E.coli	1	Shenzhen CDC	clinicai isolates

Table S2 Hybridization probe sequences for the ten pathogenic Vibros.

Type	Fluorescence channel (Tm)	Probe sequence (5′→3′)
		Detection probe: FAM-CCGGTGAAGGCTGAGCGATGAAGGACC-BHQ1
IAC	(FAM, 53.0)	L:GTGGCAGGGCGCTACGAACAATCCTACCGGTGTAGGTTGAGCCATCAAGGACCGATCGCATGACTCAGTCATCGTGAAA<br>R: GAAAGGCACAACTTTGTAGAGATTTCTGTTGAGATTGGATCTTGCTGGGC
Plesiomons shigelloides	(FAM, 57.5)	L:GTGGCAGGGCGCTACGAACAATCCTACCGGTGTAGGCTCAGCGATCAAGGACCCAACCCTTTGCAAACTCCGAATACCGTAGA<br>R: GTGCTATCCGGGAGACACACGGCGGGTTGAGATTGGATCTTGCTGGGC
Aeromonas	(FAM, 59.5)	L:GTGGCAGGGCGCTACGAACAATCCTACCGGTGATGGCTGACCGATCAAGGACCGCGGCAGCGGGAAAGTAGCTTGCTACT<br>R: TTTGCCGGCGAGCGGCGGACGGGTGAGTAATTGAGATTGGATCTTGCTGGGC
V.parahaemolyticus Tdh	(FAM, 62.5)	L:GTGGCAGGGCGCTACGAACAATCCTACCGGTGATGGCTGAGCGATTAAGGACCGGYTGACATCCTACATGACTGTGAACATTAAT<br>R: GATAAAGACTATACAATGGCAGCGGTGTCTGTGAGATTGGATCTTGCTGGGC
		Detection probe: ROX-ACGACTCTGGCTGCTCGTTCGTGACG-BHQ2
V. cholerae	(ROX, 52.5)	L:GTGGCAGGGCGCTACGAACAATCCTAACGACTATGGCTTCTCGTTGGTGACGTAGATGATGACAGCACGGGAGCCGGCATT<br>R: CATCTGAATGATCAACTCGGTTATCGTCAGTGAGATTGGATCTTGCTGGGC
V. fluvialis	(ROX, 55.5)	L:GTGGCAGGGCGCTACGAACAATCCTAACGACTCTAGCTTCTCGTTAGTGACGGCCGTCATACTGCCTATTTGTGTCATTTTGTTT

		R: ACGACGGCTTCGCAGTCTAAATTTCGATGAGATTGGATCTTGCTGGGC
V. vulnificus	(ROX, 61.5)	L:GTGGCAGGGCGCTACGAACAATCCTAACGACTCTAGCTGCTTGTTCGTGACGGAGCAACA<br>ACGATCTCTGCCTAGATGTTTAT<br>R: GGTGAGAACGGTGACAAAACGGTTGTGAGATTGGATCTTGCTGGGC
V.parahaemolyticus	(ROX, 65.5)	L:GTGGCAGGGCGCTACGAACAATCCTAACGACTCTGTCTTCTCGTTCGTGACGAACCAGAAG<br>CGCCAGTAGTACCTGAAAAAGCA<br>R: CCTGTGGCTTCTGCTGTGAATCCTTGGATTTGAGATTGGATCTTGCTGGGC
V.mimicus	(ROX, 69.5)	L:GTGGCAGGGCGCTACGAACAATCCTAACGACTCTAGCTGCTCGTTCGTGACGCACACAACA<br>TCAAGGATGACGACGGTAA<br>R: CCCACGAGAAGCATTCAAACTGGGTTTTGAGATTGGATCTTGCTGGGC
V. alginolyticus	(ROX, 72.5)	L:GTGGCAGGGCGCTACGAACAATCCTAACGACTCTGGCTGCTCGTTCGTGACGGAATGATCA<br>ATACGCCCAGCACTGGTATAT<br>R: GCGCAATGCGGTTAATGGTGTTTTCACTATTGAGATTGGATCTTGCTGGGC
		Detection probe: Cy5-CGGTGAGGCCCTTGGCAGGTTGTATCACCC-BHQ2
O1 *V. cholerae*	(CY5 55.0)	L:GTGGCAGGGCGCTACGAACAATCCTACGGTGAGGCTCTTGACAGTTTGGGATCACCCGAG<br>GAAGCAAAAATGGCTGGCTAACCAA<br>R: CCATTAGAAGCTATTCTTGGTTTAATCAATGAAGCTGAGATTGGATCTTGCTGGGC
O139 *V. cholerae*	(CY5, 62.5)	L:GTGGCAGGGCGCTACGAACAATCCTACGGTGAGGACCTTGGCAAGTTGGTATCACCCCAGA<br>TCGTGCTACGATGGCGTGTTCATTA<br>R: GAAGGGCGGGTTCCCTTGTTAGACCATGAGATTGGATCTTGCTGGGC
V. cholerae<br>CTX	(CY5, 68.5)	L:GTGGCAGGGCGCTACGAACAATCCTACGGTGAAGCCCTTGGCAGGTTGGTATCACCCGATG<br>ATGGATATGTTTCCACCTCAATTAGTTTGA<br>R: GAAGTGCCCACTTAGTGGGTCAAACTATATTGTTGAGATTGGATCTTGCTGGGC

Table S3 Sequences of primers and probes for the MLMA assay in the study

Name[a]	Sequence(5'-3')	Amount（μM）
universal primer F	GTGGCAGGGCGCTACGAACAAT	0.015μM
universal primer R	GCCCAGCAAGATCCAATCTCA	0.4μM
Detection probe FAM	FAM-CCGGTGAAGGCTGAGCGATGAAGGACC-BHQ1	0.16μM
Detection probe ROX	ROX-ACGACTCTGGCTGCTCGTTCGTGACG-BHQ2	0.08μM
Detection probe Cy5	Cy5-CGGTGAGGCCCTTGGCAGGTTGTATCACCC-BHQ2	0.16μM

[a] F: forward primer. R: reverse primer.

Table S4 Primer and probe sequences for monoplex real-time PCR assay in this study

Species	Target gene	Primer/Probe Sequence [a,]		Amplicon size (bp)	Reference
Vibrio cholerae	*hlyA*	F<br>R<br>P	ACTCGGTTATCGTCAGTTTGG<br>CGCTTTATTGTTCGATGCGTTA<br>FAM-CCCCGATAATCTTGGGCAATCGCATCGGGG-BHQ1	141	[1]
Vibrio cholerae	*CTX*	F<br>R<br>P	TCCGGAGCATAGAGCTTGGA<br>TCGATGATCTTGGAGCATTCC<br>FAM- -CCGTGGATTCATCATGCACCGCCACGG -BHQ1	120	[1]
Vibrio parahemolyticus	*toxR*	F<br>R<br>P	AAGCGCCAGTAGTACCTGA<br>CCAATCTGACGGAACTGAGATT<br>FAM- CGGCAAATCGGTAGTAATAGTGCCG -BHQ1	185	[2]
Vibrio parahemolyticus	*tdh*	F<br>R<br>P	AAACATTTGCCTTTGAGCTTCCA<br>CTCGAACAACAAACAATATCTCATCAG<br>FAM- CCGGGGTGTCCCTTTTCCTGCCCCCGG-BHQ1	74	[3]
Vibrio fluvialis	*toxR*	F<br>R<br>P	GACGCTTGGCAGTGTTCAAC<br>GTGCATTCCACCATATTTTCTTACG<br>FAM-TGTCAGCACGCCAATCAATCACCCG-BHQ1	113	[4]

[a]F, R and P represent forward primer, reverse primer and detection probe, respectively.

References

1. Huang, J., et al., *Quadruplex Real-Time PCR Assay for Detection and Identification of Vibrio cholerae O1 and O139 Strains and Determination of Their Toxigenic Potential.* Applied and Environmental Microbiology, 2009. **75**(22): p. 6981-6985.

2. Hu, Q., et al., *A Modified Molecular Beacons–Based Multiplex Real-Time PCR Assay for Simultaneous Detection of Eight Foodborne Pathogens in a Single Reaction and Its Application.* Foodborne Pathogens and Disease, 2014. **11**(3): p. 207-214.

3. Blackstone, G.M., et al., *Detection of pathogenic Vibrio parahaemolyticus in oyster enrichments by real time PCR.* Journal of Microbiological Methods, 2003. **53**(2): p. 149-155.

4 Jia, Q., et al., *Detection of Vibrio fluvialis with Taqman real time PCR assay. Chinese Journal of Public Health*, 2011. **27**(3): P. 378-380. In Chinese.

Table S5 The artificial tag sequences with unique Tm values in three fluorescent channels

Fluorescence	The label sequence	Temperature(Tm ℃)
ROX	ROX-ACGACTCTGGCTGCTCGTTCGTGACG-BHQ2	
	ACGACTCTGGCTGCTCGTTCGTGACG	72.5
	ACGACTCTAGCTGCTCGTTCGTGACG	69.5
	ACGACTCTAGCTGCTTGTTCGTGACG	65.5
	ACGACTCTAGCTTCTCGTTAGTGACG	61.5
	ACGACTCTGTCTTCTCGTTCGTGACG	55.5
	ACGACTATGGCTTCTCGTTGGTGACG	52.5
FAM	FAM-CCGGTGAAGGCTGAGCGATGAAGGACC-BHQ1	
	CCGGTGATGGCTGAGCGATTAAGGACC	62.5
	CCGGTGTAGGCTCAGCGATCAAGGACC	59.5
	CCGGTGATGGCTGACCGATCAAGGACC	57.5
	CCGGTGTAGGTTGAGCCATCAAGGACC	53
Cy5	Cy5-CGGTGAGGCCCTTGGCAGGTTGTATCACCC-BHQ2	
	CGGTGAGGCTCTTGACAGTTTGGGATCACCC	55
	CGGTGAGGACCTTGGCAAGTTGGTATCACCC	62.5
	CGGTGAAGCCCTTGGCAGGTTGGTATCACCC	68

Table S6 The discrepant results between the MLMA assay and conventional method

**No.**	**Sample ID**	conventional method	the MLMA assay	Singleplex real-time PCR Ct value
1	SZ210	ND	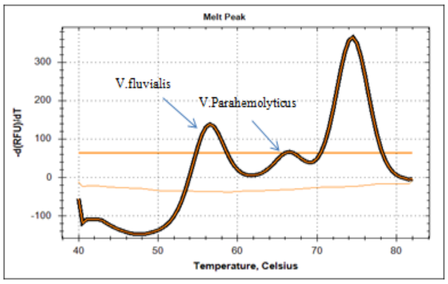	18 (V.fluvialis）<br>21 (V.Parahemolyticus)
2	SZ229	ND	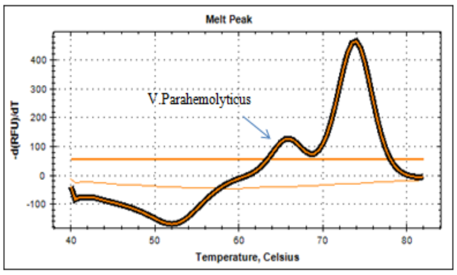	19 (V.Parahemolyticus)
3	SZ248	ND	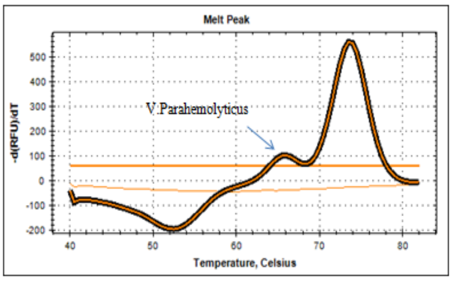	21 (V.Parahemolyticus)
4	SZ254	ND	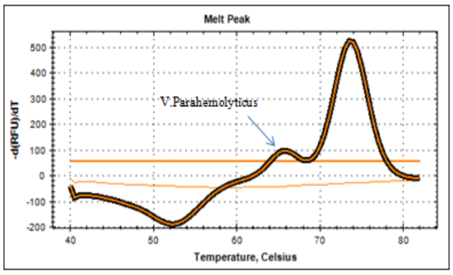	23 (V.Parahemolyticus)
5	SZ261	ND	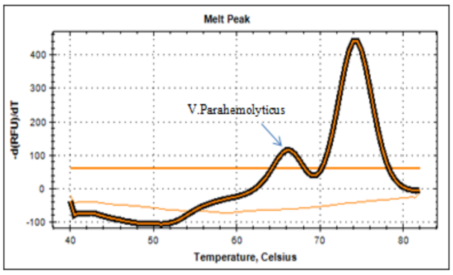	19 (V.Parahemolyticus)
6	SZ263	ND	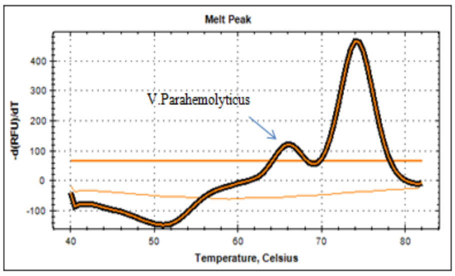	18 (V.Parahemolyticus)

ND: not detected

Figure S7 Simultaneous identification of pathogenic Vibrios in mixed infection

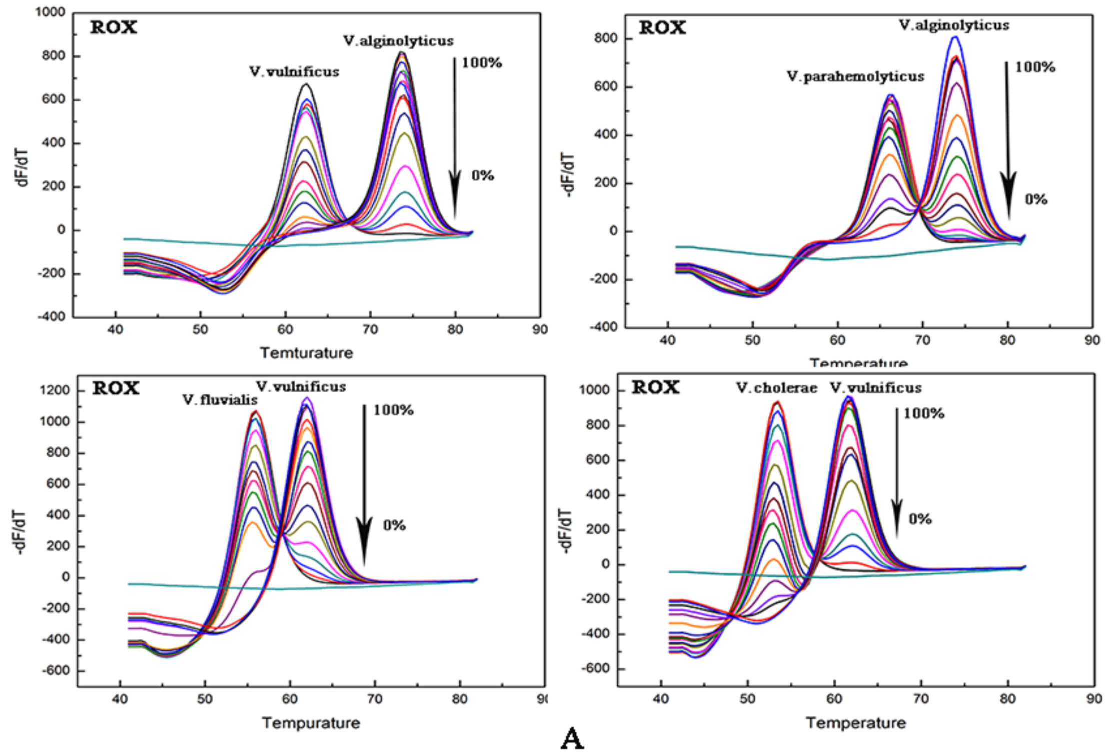

A

Figure A: *V. vulnificus* and *V.alginolyticus*, *V. parahaemolyticus* and *V.alginolyticus*, *V. fluvialis* and *V. vulnificus*, *V. cholera* and *V. vulnificus* were identified in the same fluorescence channel, The MLMA assay was performed by various percentage ratios of the two species (100:0, 97:3, 95:5, 90:10, 80:20, 70:30,60:40, 50:50, 40:60, 30:70,20:80, 10:90, 5:95, 3:97, 0:100), and DEPC water was used as a negative control.

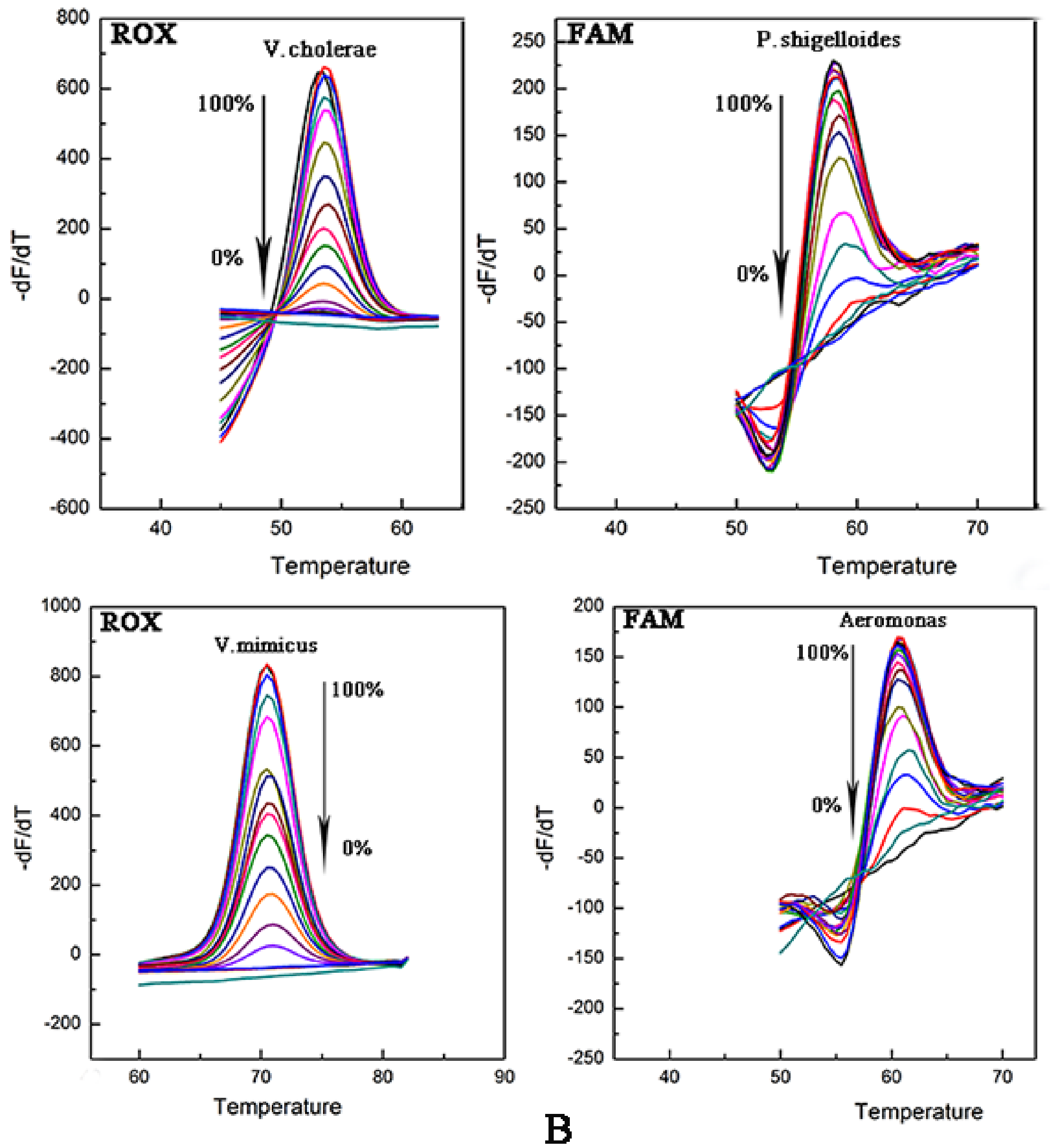

B

Figure B: *V. cholera* and *P. shigelloides*, *V.mimicus* and *Aeromonas* were identified in the same fluorescence channel, The MLMA assay was performed by various percentage ratios of the two species (100:0, 97:3, 95:5, 90:10, 80:20, 70:30,60:40, 50:50, 40:60, 30:70,20:80, 10:90, 5:95, 3:97, 0:100), and DEPC water was used as a negative control.
